# Assessment of knowledge and acceptability of HIV self-testing among students of selected universities in southwest Nigeria: an online cross-sectional study

**DOI:** 10.11604/pamj.2022.43.94.31741

**Published:** 2022-10-24

**Authors:** Abdulhammed Opeyemi Babatunde, Progress Agboola, Yusuf Babatunde, Esther Bosede Ilesanmi, Habibllah Ayodele, Oliver Chukwujekwu Ezechi

**Affiliations:** 1 Medicine and Surgery, Faculty of Clinical Sciences, College of Medicine, University of Ibadan, Ibadan, Nigeria,; 2 Healthy Africans Platform, Ibadan, Nigeria,; 3 Department of Medicine and Surgery, Faculty of Clinical Sciences, Ladoke Akintola University of Technology, Ogbomoso, Nigeria,; 4 Department of Pharmacy, University of Ilorin, Ilorin, Kwara State, Nigeria,; 5 Department of Nursing Services, University College Hospital, Ibadan, Oyo State, Nigeria,; 6 Department of Mechanical Engineering, Faculty of Technology, University of Ibadan, Ibadan, Nigeria,; 7 Centre for Reproductive and Population Health Studies, Lagos, Nigeria,; 8 Nigerian Institute of Medical Research, Yaba, Lagos, Nigeria; 9 Department of Public Health, Lead City University, Ibadan, Nigeria

**Keywords:** Nigeria, students, university, HIV/AIDs

## Abstract

**Introduction:**

in Nigeria, it was estimated that 1.9 million people were living with HIV of which 130,000 people were newly infected with HIV. HIV self-testing would potentially increase access to HIV testing for people to know their status, get diagnosed, and initiate treatment as soon as possible. Our study aims to assess the knowledge of HIV Self-Testing (HIVST) and the acceptability of this youth-friendly approach among students in southwest Nigeria online.

**Methods:**

a cross-sectional study was conducted among bona fide undergraduate students (2019/2020 session) of two popular tertiary institutions in southwest Nigeria. An online standardized self-administered questionnaire was administered using Google Forms. Microsoft Excel and IBM SPSS statistics were used for tabulation and statistical data analysis. The Chi-Square test was conducted using a P value of 0.05 to determine the level of significance.

**Results:**

of the 155 students that participated in the study, 82 (52.9%) were male. Most of the respondents (65.2%) were studying medicine and other health-related courses. The mean knowledge of HIVST among respondents was slightly above average. Respondents studying medical and other health-related courses showed a slightly better level of knowledge than others although not statistically significant (P = 0.222). 76.1% of respondents had never used the HIVST option before and 62.6% are willing to use it sometimes in the future.

**Conclusion:**

to achieve the UNAIDS 95-95-95 fast-track targets in Nigeria by 2030, there is a need to promote sexual and reproductive health education and increase awareness and accessibility of HIVST to youths.

## Introduction

It was estimated in 2017 that 36.9 million (31.1-43.9 million) persons were living with HIV and 21.7 million (19.1-22.6 million) were getting access to antiretroviral therapy (ART) worldwide [1]. The Joint United Nations Programme on HIV/AIDS (UNAIDS) has introduced a formidable global 95-95-95 target to be achieved by 2030. The goal is to reach 95% of adults who know their HIV status, 95% of those who are HIV positive are accessing ART services, and 95% of those enrolled in ART have viral suppression by 2030. However, the usual HIV testing services (HTS) are not effective and adequate to attain universal access to HIV prevention, treatment, and management, especially for those that have not been tested, those at higher risk of contracting HIV, and marginalized populations [1].

In sub-Saharan Africa, only 40% of people living with HIV (PLHIV) know their HIV status even with the availability of HIV testing service centres across the regions [1]. Nigeria has the second-highest number of PLHIV who are unaware of their status. In Nigeria in 2018, it was estimated that 1.9 million people were living with HIV of which 130,000 people were newly infected with HIV and 53,000 people died from an AIDS-related illness [2]. Out of the confirmed population, 67% of people living with HIV knew their status, 53% of people living with HIV were on treatment and 42% of people living with HIV were virally suppressed [2].

To meet UNAIDS ambitious goal, there is a need for increased HIV testing and ensuring that no one is left behind. The concept of HIV self-testing is to make HIV testing more convenient and ensure privacy through a youth-friendly approach. There have been concerns about stigma and confidentiality which limits the uptake of HIV testing among young adults [3]. Also, for a key population of groups including men who have sex with men (MSM), female sex workers, and injecting drug users, the uptake of HIV testing services is often reduced due to several factors such as stigma and discrimination, criminalization, and fear of having trouble with law enforcement authorities [3].

HIV self-testing can increase the number of people living with HIV who have access to testing, know their status, are diagnosed, and initiate treatment as soon as possible [4]. To overcome barriers to HIV testing uptake, the World Health Organization (WHO) developed HIV guidelines recommending HIV self-testing (HIVST) be offered in addition to existing facility-testing modalities (2016) [5]. Oral HIVST involves the person obtaining an oral sample by swiping the gums with the appropriate test swab, doing the test, and analysing the test's result privately. It is said to be a screening test and does not indicate a definitive diagnosis; thus, it requires confirmative testing [3].

The HIVST policy landscape is changing rapidly. Following the release of the WHO guidelines in 2016, many countries including Nigeria have been quick to take up HIVST national policies and have already begun to implement various approaches [6]. In considering the need to adopt these innovative approaches to ensure more people access HIV diagnosis and get treatment, HIVST remains an effective new way to reach more people than what is currently available through normal facility visits and testing. Some quantitative studies have been conducted in other African countries to access the acceptability and feasibility of HIVST among young people in Africa, however, none has been carried out in Nigeria [7-10]. Although a recent study evaluated the preference for HIVST among young Nigerians using a qualitative approach [11].

Our study aims to assess the knowledge of HIV self-testing and the acceptability of this youth-friendly approach among undergraduate students in southwest Nigeria. This study also assesses the relationship between the knowledge of HIV self-testing and the socio-demographic characteristics of students.

## Methods

**Study design:** this study employed a cross-sectional study design among current undergraduate students of two popular tertiary institutions in southwest Nigeria - the University of Ibadan, Ibadan, Nigeria, and the Ladoke Akintola University of Technology, Ogbomoso, Nigeria

**Study setting:** Nigeria is the most populous country in Africa, with an estimated population of 200 million and about 1.8 million people living with HIV. South West Nigeria is one of the geopolitical zones of Nigeria consisting of six States and is mainly populated by the Yoruba tribe. The University of Ibadan and the Ladoke Akintola University of Technology are two major universities in this region with about 30,000 students each.

**Sample size estimation:** the convenient sampling technique was adopted in the study. No prior sample size was calculated. The survey was online-based and required the voluntary participation of eligible students with access to the internet.

**Study instrument and administration:** the study questionnaire was developed from previous studies on knowledge and willingness to use HIVST and modified by the researchers to fit the objectives of this study [12,13]. Next, a pre-test was conducted by members of the research team to examine for readability, comprehensibility and grammatical error. The final questionnaire was validated by Sexual and Reproductive Health experts comprising sections on socio-demographic characteristics, awareness, and knowledge of HIVST, and acceptability of HIVST. The final questionnaire was then used to develop the online survey using Google form and a link to the e-survey was shared.

**Study participants:** study participants included all current undergraduate students of the University of Ibadan, Ibadan, Nigeria, and the Ladoke Akintola University of Technology, Ogbomoso, Nigeria with access to the internet. A convenient sampling technique was used. A standardized self-administered questionnaire was designed using google forms and the link to the questionnaire was sent to students and student groups of both universities via WhatsApp and Facebook. The voluntary response and snowball sampling techniques were adopted. Eligible respondents were encouraged to send the link to other prospective respondents on their WhatsApp contact list and share it on other online platforms. The standardized questionnaire conformed to the Helsinki Declaration, the purpose of the study was explained to each participant and informed consent was provided on the first page of the questionnaire where a participant could willingly choose whether or not to participate in the study. The online questionnaire was employed as a result of the closure of institutions as an intervention to mitigate the spread of the COVID-19 pandemic in the country. Besides, the study population is students who are most active on the internet, have access to mobile phones, and also understand the English language. The study was carried out over one month in September 2020. A total of 155 students were enrolled.

**Study variables and data sources:** the online questionnaire used in this study was drafted in English Language and piloted among 10 members of the research team for validation before sharing online. It comprised of three sections which included: socio-demographic characteristics, the knowledge and awareness of HIV-Self-Testing, and acceptability of HIV-Self-Testing. The socio-demographic variables included gender, age, marital status, level of study, and type of student (whether medical or health-related student or not). The level of knowledge and awareness of HIV-Self-Testing was determined through a five-point Likert-type scale about whether the respondents “strongly disagree”, “disagree”, “neutral”, “agree”, or “strongly agree” and consisted of twelve (12) items, eight of which tested for knowledge while others tested for awareness. Knowledge items were given a score of 1 for “strongly agree” or “agree” while “neutral”, “disagree” or “strongly disagree” was given a score of 0 for a positively phrased question and vice versa for negatively phrased questions. Total knowledge score (range = 0 to 8) were arbitrarily categorized into 0-2 = poor, 3-5 = average, and 6-8 = good. Level of acceptability of HIV Self-Testing comprised of eight (8) items answered as either “Yes”, “No” or “I don't know”.

**Bias:** potential sources of bias for this study were from the method of data collection which was online. This was controlled for, in the study design by including the name of schools and simple questions about the school which is expected to be known by all students of the respective schools. Also, the links were shared through student platforms and a study was conducted for a short time to reduce spam.

**Quantitative variables and statistical methods:** the data collected online was saved to Microsoft Excel for cleaning and re-coding and then exported to IBM SPSS Statistics version 20 for tabulation and statistical data analysis. Descriptive analysis for frequency and inferential statistics with the Chi-Square test was conducted. A P value of 0.05 at a 95% confidence interval was used to determine the level of significance of the variables.

**Ethical consideration:** the study was conducted during the COVID-19 lockdown and no formal ethical approval was collected from the institution's ethical review body. However, we adhered strictly to all the ethical issues involved in conducting studies involving human participants such as informed consent which was obtained electronically and clearly stated participants' rights to withdraw anytime from the study. Besides, we ensured confidentiality and anonymity by not requesting any personal or identifiable codes on the online survey.

## Results

**Participants:** the total number of responses recorded was 156 while only the data from 155 respondents who consented to participate in the study was included in the analysis.

**Descriptive data:** of the 155 students that participated in the study, 82 (52.9%) were male. The majority (58.1%) were within the age range of 21-25 years old. The majority (64.5%) of the participants were students from the University of Ibadan. Most of the respondents (65.2%) were studying medicine and other health-related courses. Table 1 shows the socio-demographic characteristics of respondents.

**Table 1:** socio-demographic characteristics of respondents

	n =155	%
**Gender**		
Male	82	52.9
Female	72	46.5
Prefer not to say	1	0.6
**Age**		
16-20	46	29.7
21-25	90	58.1
26-30	18	11.6
>30	1	0.6
**Marital status**		
Single	147	94.8
Married	8	5.2
**Institution**		
University of Ibadan	100	64.5
Ladoke Akintola University of Technology	55	35.5
**Course**		
Medical and health-related	101	65.2
Non-health-related	54	34.8
**Level**		
100 level	13	8.4
200 level	27	17.4
300 level	39	25.2
400 level	47	30.3
500 level	22	14.2
600 level	7	4.5

**Figure 1:**
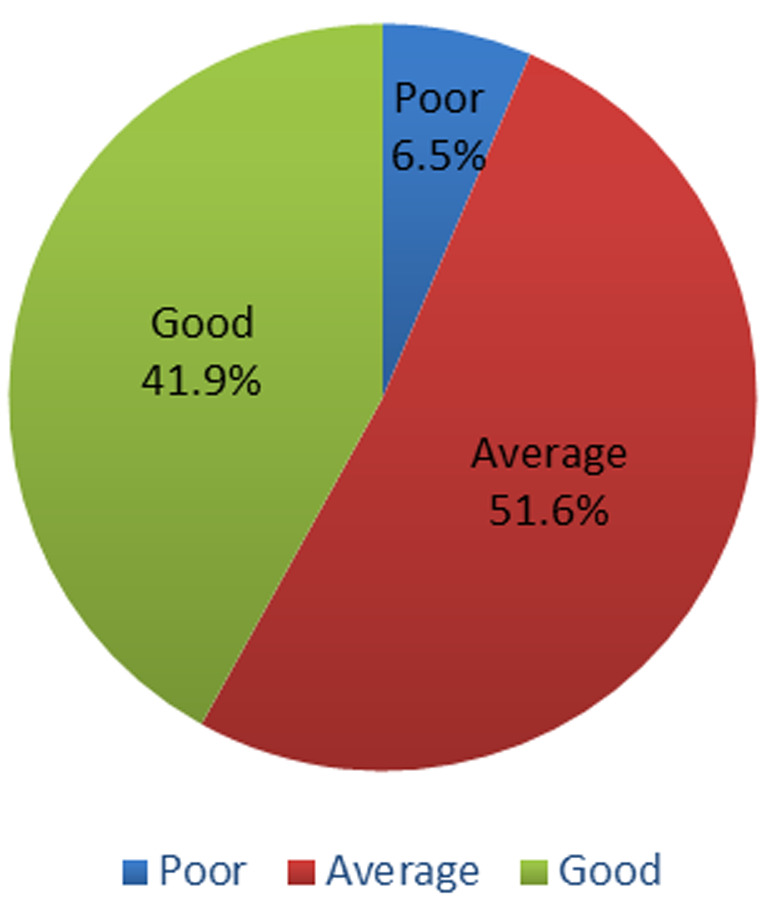
level of knowledge of HIVST among respondents

**Outcome data:** about 6.5% of the respondents had poor knowledge of HIVST (Figure 1). The mean knowledge of HIVST among respondents was slightly above average (mean = 5.1, median = 5, SD = 1.4). Most of the respondents gave a wrong answer to the question testing their understanding of the principle guiding HIVST kit about the reactivity of antibodies with reagents. Only 36% understood that HIVST kits were not reusable. 94.2% of respondents were aware of the need for a confirmatory diagnostic test in the hospital if tested positive with an HIVST kit (Table 2). About seventy-six percent (76.1%) of respondents have never used the HIVST option before and 62.6% are willing to use it sometimes in the future. Most respondents prefer HIV counselling and testing (HCT) to HIVST (52.3% and 32.3% respectively). However, there is average general acceptability (56.9%) of HIVST (Table 3). There was no statistically significant difference between the level of knowledge and any of the socio-demographic characteristics although respondents studying medical and other health-related courses showed a slightly better level of knowledge than others (P = 0.222).

**Table 2:** selected items measuring knowledge on HIV self-testing (HIVST)

1.	To what extent do you agree to the following?	Strongly disagree	Disagree	Neutral	Agree	Strongly Agree
2.	Can I use an HIVST kit more than once?	20(12.9)*	29(18.7)*	86(55.5)	12(7.7)	8(5.2)
3.	Can HIVST be done on blood samples?	2(1.3)	3(1.9)	9(5.8)	46(29.7)*	95(61.3)*
4.	Can HIVST be done on oral fluid samples?	4(2.6)	18(11.6)	51(32.9)	52(33.5)*	30(19.4)*
5.	Will HIVST results be available after 20 to 40 minutes?	1(0.6)	5(3.2)	45(29.0)	53(34.2)*	51(32.9)*
6.	Is a confirmatory test required if the test result came out positive?	1(0.6)	3(1.9)	5(3.2)	42(27.1)*	104(67.1)*
7.	Is a repeated HIVST required after 3 months of last exposure if tested negative?	2(1.3)	18(11.6)	52(33.5)	83(53.5)*	0(0.0)*
8.	Can a rapid HIVST be done at home?	2(1.3)	10(6.5)	35(22.6)	61(39.4)*	47(30.3)*
9.	Do reactive results mean that the test indicated that antibodies are absent in the oral fluid sample?	4(2.6)*	14(9.0)*	85(54.8)	28(18.1)	24(15.5)

* Correct response

**Table 3:** selected items on the level of acceptability of HIV self-testing

		Frequency (percentage)		
	Do you agree with the following	No	I don't know	Yes
1.	Can you use the HIVST option?	31(20.0)	27(17.4)	97(62.6)
2.	Will you like to substitute HCT with HIVST?	81(52.3)	27(17.4)	47(30.3)
3.	Will you like your partner to get tested using the HIVST option?	38(24.5)	22(14.2)	95(61.3)
4.	Will you like to check the results of HIVST yourself?	18(11.6)	12(7.7)	125(80.6)
5.	Will you be nervous while reading your HIVST result?	54(34.8)	15(9.7)	86(55.5)
6.	Will you be ready to pay for your HIVST kit?	32(20.6)	22(14.2)	101(65.2)
7.	Will you visit the hospital for treatment if you tested positive using HIVST?	14(9.0)	12(7.7)	129(83.2)
8	Have you ever used HIVST?	118(76.1)	11(7.1)	26(16.8)
	Total	386 (31.1)	148 (11.9)	706 (56.9)

HCT HIV Counseling and Testing

## Discussion

This study investigated the knowledge and acceptability of HIV self-testing (HIVST) amongst students of selected universities in southern Nigeria. Majority of the respondents were youths and single. From our results, there was no considerable difference between the level of knowledge and any of the socio-demographic characteristics. However, respondents studying medical and other health-related courses showed a slightly better level of knowledge than others. The findings of this study revealed average knowledge (51.6%) and high acceptability (62.6%) of HIVST among students of selected universities in southern Nigeria. The results from our study had similar proportions in terms of knowledge of HIVST in other African institutions [14,15]. Although, the level of acceptability was higher in other parts of Africa (70% - 87.1%) [14-21], and Canada (81%) [22]. The lower acceptance among our respondents when compared to other studies across Africa and Canada could be because of variations in prevalence, the intensity of HIV interventions, and cultural differences as well as incentives [23-26].

Despite the high level of acceptability of HIVST, most students had concerns and preferred HIV counselling and testing (HCT) to HIVST (52.3% and 32.3% respectively). The possible reasons were not investigated in this study. These concerns were in sync with a study conducted in Tanzania to determine the level of knowledge, acceptability, and willingness to use oral fluid HIV self-testing among medical students. Findings from the study revealed that lack of pre and post-test counselling after self-testing was a key concern among the respondents [27]. These concerns also concur with findings from different settings highlighting the importance of HCT and HIVST [28-34].

In addition, 65.2% of the respondents were ready to pay for the HIVST kit. A previous study conducted has reported that the cost of purchasing a self-test kit is one of the major barriers that hinder willingness to use and conduct HIVST [28,29,33,35,36]. Our study was in sync with many other studies assessing willingness to HIVST in other different settings [37-39] but contrary to the findings from Hlongwa *et al*. among medical students in Tanzania [27]. The reason for the contrary result could be because of the economical differences between Nigeria and Tanzania [27]. This comparison is really important in addressing the issue of the cost of acquiring these HIVST kits, particularly among young people in low-income countries, to increase the uptake of HIVST. According to Charlene *et al*., in sub-Saharan Africa, the cost to purchase HIVST kits will likely compete with daily necessities such as food due to high levels of poverty when compared to high-income countries [40]. Global evidence has suggested that HIVST has the potential to overcome the existing barriers with existing HIV testing interventions [41,42]. Barriers such as fear of stigma and discrimination, lack of privacy and confidentiality, and hard-to-reach key populations affect already existing HIV testing services [37-41]. It is therefore key to develop interventions that ensure proper awareness of HIVST to all populations and ensure HIVST and its concept are incorporated into the pre-service curriculum for medical and health-related courses. There is a high potential for HIVST to reach key populations at high risk of HIV/AIDS and this means specific measures and innovations like the cost of the self-kits, delivering the kits to people in the comfort of their homes and much more has to be addressed to ensure HIVST is acceptable by all. **Limitations:** the study aimed at assessing the knowledge and acceptability of HIVST among students of selected Universities. However, some limitations and sources of biases were identified. Due to the sampling method used in data collection, most of the respondents were students from Health Sciences who are expected to have basic knowledge of HIVST. Another limitation of our study is the sample size but it covers a key insight into the knowledge and acceptability of HIVST. Also, based on the convenient sampling technique used and the method of data collection which is online, our study could have omitted some students with limited or no access to the internet hence, the results may not represent the general population.

## Conclusion

Our study indicates average knowledge and moderate acceptability of HIVST among the selected students without any significant association with socio-demographic characteristics. However, there is a need to promote awareness about HIVST among youths in Nigeria to achieve the UNAIDS 95-95-95 fast-track targets by 2030 [42].

### What is known about this topic

- More than half of people living with HIV in Africa do not know their status;
- HIV prevalence is high in Nigeria, especially among the youths.

### What this study adds

- University students have an average level of knowledge about HIV Self-Testing;
- HIV Self-Testing has the potential to be acceptable by University students;
- There is no significant association between socio-demographic characteristics and the level of knowledge about HIV Self-Testing among participants.
